# Use of tetrasodium EDTA acid for the treatment of intraluminal obstruction of subcutaneous ureteral bypass devices

**DOI:** 10.1177/1098612X221107795

**Published:** 2022-06-28

**Authors:** Valerie Duval, Marilyn Dunn, Catherine Vachon

**Affiliations:** Department of Clinical Sciences, Faculty of Veterinary Medicine, University of Montréal, Saint-Hyacinthe, QC, Canada

**Keywords:** Tetrasodium ethylenediaminetetraacetic acid, ureteral obstruction, subcutaneous ureteral bypass, calcium oxalate stone

## Abstract

**Objectives:**

The aim of this study was to evaluate the efficacy and tolerability of a 4% tetrasodium EDTA (tEDTA) infusion protocol in the subcutaneous ureteral bypass (SUB) devices of cats with intraluminal obstruction at a veterinary teaching hospital between July 2017 and April 2020.

**Methods:**

This was a retrospective controlled study. Cats with an obstructed SUB device underwent a 4% tEDTA infusion protocol. Obstruction of the device was diagnosed based on renal pelvic dilation, dilatation of the ureter, mineralized material within the device (cystostomy or nephrostomy catheters) seen on ultrasound, the absence of visible bubbles within the renal pelvis and/or urinary bladder following ultrasound-guided flushing of the device with saline.

**Results:**

A total of 16 tEDTA infusion protocols were performed in 14 cats. The infusion protocol was considered successful in 11/16 SUB devices (68.8%). Six devices (n = 6/11; 54.5%) had recurrence of obstruction with a median time of 87 days. One or more episodes of self-limiting pollakiuria and/or hematuria following infusion was seen in eight patients (n = 8/14; 57.1%).

**Conclusions and relevance:**

Infusions of 4% tEDTA successfully relieved intraluminal obstruction in patients with occluded SUB devices; however, the recurrence of obstruction was common. Additional studies evaluating case selection and optimal protocols are warranted.

## Introduction

Placement of a subcutaneous ureteral bypass (SUB) device has been associated with positive outcomes for the treatment of benign feline ureteral obstruction. Despite their success, SUB devices have been associated with some long-term complications. Intraluminal obstruction of the device with mineralized debris has been reported in 24% of patients and the median time to intraluminal obstruction was 463 days in one study.^1^ Surgical catheter replacement is required to restore patency in patients with intraluminal SUB device obstruction and concurrent ongoing ureteral obstruction (dilated renal pelvis, dilated ureter ± increasing creatinine). In a recent retrospective study, only 5/39 mineralized SUB devices (12.8%) required exchange; ipsilateral ureteral patency was re-established in the remaining patients.^1^

Feline upper urinary tract stones cannot be dissolved medically as they are mainly composed of calcium oxalate (CaOx).^2,3^ The authors have submitted sand and small stones from the catheters of cats with intraluminal obstruction of their SUB devices and confirmed that the obstructive mineral debris was composed of both mono and dihydrate CaOx.

In the human literature, studies have shown that tetrasodium EDTA (tEDTA) has the ability to chelate calcium within CaOx stones and therefore result in their dissolution in vitro.^4–6^ EDTA is a metalloprotease inhibitor that acts as a chelating agent. It has the ability to form stable complexes in solution with multiple ions such as magnesium, calcium, strontium and barium, and therefore is able to dissolve CaOx crystals.^7^ In people, tEDTA has been shown to eradicate biofilm in central venous catheters^8^ and from wounds.^9^ It is used in cosmetics,^10^ to treat lead and mercury intoxication,^11^ and has been proven to be helpful in treating some vascular conditions^11^ and in reducing blood cholesterol.^10^ Furthermore, its use in kidney stone dissolution and chemolysis has been investigated in humans.^12^

A recent study assessing the use of a 2% tEDTA^13^ solution (Norfolk Vet Products) for the treatment of mineral occlusion in eight SUB devices (six cats) showed restored patency in all eight SUB devices infused with tEDTA. Infusions of 2% tEDTA into SUB devices were well tolerated and associated with stable-to-improved creatinine levels. A recent abstract reported a decreased rate of bacterial infection, mineralization and the need for device exchange when 2% tEDTA was used as a locking solution following prophylactic SUB device flushing when compared with flushes performed with mixed saline/tEDTA or saline.^14^

The aim of this study was to evaluate the efficacy and tolerability of a 4% tEDTA infusion protocol in SUB devices of cats with intraluminal obstruction. Our main hypotheses were that (1) performing a tEDTA protocol would restore SUB device patency and prolong the interval between SUB device obstruction and the need for surgical catheter replacement or euthanasia; and (2) that an infusion of 4% tEDTA into the SUB device would be well tolerated.

## Materials and methods

### Study design and case selection

A retrospective controlled study was carried out between July 2017 and April 2020 on cats presented to the Centre Hospitalier Universitaire de Montréal with an obstructed SUB device and having undergone a 4% tEDTA infusion protocol. Cases were included when obstruction of the SUB device and concurrent ureteral obstruction were diagnosed based on renal pelvic and ureteral dilation as compared with previous measurements during routine SUB device flushing with patent catheters, mineralized material within the SUB device (cystostomy or nephrostomy catheters) seen on ultrasound with absence of visible bubbles within the renal pelvis and/or urinary bladder following ultrasound-guided flushing of the SUB device. Abdominal radiographs and/or fluoroscopy were performed in order to exclude a static or positional kink, and to ensure that SUB device migration had not occurred.

### History, clinicopathologic results and data collection

Signalment, history (including cause of ureteral obstruction, arterial blood pressure, serum creatinine [at the time of SUB device placement], hematocrit, ionized calcium, serum biochemistry [including thyroid hormone] and urine culture) and imaging findings (ultrasound, fluoroscopy, radiographs) were recorded. At the first obstructive event, the following information was recorded: location of the obstruction (cystostomy catheter, nephrostomy catheter, both or subcutaneous port); reason for presentation, serum creatinine at the time of the protocol, renal pelvis measurements, the number of tEDTA infusions administered, duration of hospitalization, the number of active days (ie, numbers of days a SUB device flush was performed during a tEDTA protocol, excluding the days no flush was performed), success of the tEDTA protocol, recurrent obstructive events, survival and complications. An ‘extension time’ was also recorded and defined as the number of days following tEDTA infusion that the patient’s SUB device remained patent until surgical catheter exchange, death/euthanasia or the end of the study occurred. In this study, stable azotemia was defined as a creatinine value increasing by <30% from baseline in International Renal Interest Society (IRIS) stages 1–2 and <10% in IRIS stages 3–4.^15^

### Flush technique (irrigation technique)

A standard technique was used for all SUB device flushes. Patients were placed in dorsal recumbency, the skin over the port was shaved and aseptically prepared. A 22 G 3/4” Huber needle (Norfolk Vet Products) was connected to a 12” extension set (volume of 0.14 ml per 12”), a three-way stopcock (Smiths Medical) and two 5 ml syringes (one with 5 ml of saline and the other empty). Both syringes were connected to the three-way valve. Upon entering the port with the Huber needle, 1.0–3.0 ml urine was withdrawn. The flush was then performed by a swift injection of 0.5–1.0 ml aliquots of saline into the port while visualizing the kidney on ultrasound. Again, 0.5–1.0 ml were withdrawn from the port and a further 0.5 ml saline was injected with a swift injection while visualizing the bladder on ultrasound. All images were recorded for future reference. Patients were determined to be completely obstructed if no flush could be visualized either in the kidney or bladder or in both. A partial obstruction was determined based on decreased or delayed visualization of the flush in either the kidney or bladder or both. In some obstructed patients, aspiration from the port was impossible or difficult. If injection of tEDTA was not possible, the patient was not included in the study. After the SUB device flush, the litter box was removed from the patient’s cage for 1 h.

### tEDTA protocol

The tEDTA solution (KiteLock 4%; SterileCare) was infused through the SUB port while watching the kidney on ultrasound to ensure overdistension of the renal pelvis and calyces did not occur. If renal pelvic dilation was observed, the infusion was stopped. KiteLock 4% has a pH of 10–10.8 and an osmolality ranging from 278 to 347 mOsm/l. The solution is composed of EDTA, sterile water and sodium hydroxide for pH regulation. The solution of 2% tEDTA was made by adding an equal part of sterile saline and 4% tEDTA. The pH of the 2% tEDTA was measured and was found to be 10.12. The 2% tEDTA solution was used in patients when lower urinary tract signs (LUTS) were noted during the protocol.

Based on a protocol described by Norfolk Medical, a standard infusion protocol was used (Table 1). The protocol was adapted on an individual basis according to the patient’s response and owner’s schedule and financial constraints. At each visit, a focused urinary tract ultrasound and SUB device flush were performed. The renal pelvis and the bladder were completely emptied, if possible. A SUB device flush was performed as described above to document nephrostomy and cystostomy catheter patency. Under ultrasound guidance, 3 ml 4% tEDTA was infused into the SUB device. This was followed by an infusion of 0.2 ml sterile saline to flush the extension set.

**Table 1:** 4% tetrasodium EDTA infusion protocol along with imaging and laboratory tests performed at various time intervals

Week	Day	US-guided SUB device flush	Diagnostic procedures
*1*	1 3, 4 or 5	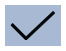	UA, UC, CHEM, Hct, iCa Ø
*2*	8	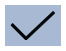	Ø
*4*	22, 23 or 24	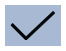	Ø
*8*	50, 51 or 52	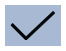	Ø
*14*	92, 93 or 94	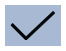	RP, UA, UC
*26*	176, 177 or 178	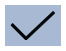	RP, UA, UC
*q3months*		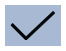	RP (CBC/CHEM q6months), UA, UC, T4 (cats >7 years old)

US = ultrasound; SUB = subcutaneous ureteral bypass; UA = urinalysis; UC = urine culture; CHEM = serum chemistry; Hct = hematocrit; iCa = ionized calcium; Ø = none; RP = renal panel; CBC = complete blood count; T4 = thyroid hormone

Depending on SUB device patency, the protocol was adjusted. If the renal pelvis, ureter or calyces remained dilated and the SUB device was completely obstructed, 4% tEDTA infusions were performed twice daily for three consecutive days. If the SUB device was partially occluded with mild pelvic dilation, two infusions of 4% tEDTA were performed once a day for two consecutive days for the first week, then weekly, then every other week, etc (as described in Table 1). A successful tEDTA protocol was defined as resolution mineralization of the SUB device sufficiently in order to regain patency (visible bubbles within the renal pelvis and/or urinary bladder following ultrasound-guided flushing), whereas an unsuccessful protocol was defined as a failure to achieve SUB device patency (absence of visible bubbles within the renal pelvis and/or urinary bladder following ultrasound-guided flushing).

### Statistical analysis

Owing to the small number of cases in this study, descriptive statistics (means, ranges and medians) were performed.

## Results

### Patient signalment

Between July 2017 and April 2020, 14 cats underwent tEDTA infusion protocols for a total of 16 obstructed SUB devices (Table 2). The 14 cats consisted of eight spayed females and six castrated males. The population included 13 domestic shorthairs and one Cornish Rex. The median age and weight at the time of SUB device placement were 8.9 years (range 3–11.7; mean 8.4) and 5.11 kg (range 2.21–6.11; mean 4.65), respectively. Ureterolithiasis was the cause of 14 obstructive events (n = 14/16; 87.5%). The two remaining obstructive events (12.5%) were suspected to be secondary to a ureteral stricture. Overall, nine cats had bilateral and five cats had unilateral SUB devices. Median time between SUB device placement and the first obstructive event was 405 days (range 90–1514; mean 431.2). The median number of routine SUB device flushes performed prior to the first obstructive event was four (range 0–18; mean 5.3).

**Table 2:** 4% tetrasodium EDTA infusion protocol results and outcomes in 14 cats and 16 subcutaneous ureteral bypass (SUB) devices

Patient	SUB	SUB information	First obstruction event	Outcome	# infusion (first event)	Extension time (days)
A	SUB#1	Unilateral, left	Left nephrostomy; partial obstruction	S (two recurrences)	9	134
B	SUB#2	Bilateral	Left nephrostomy; partial obstruction	S (no recurrence)	15	140*
C	SUB#3	Unilateral, left	Left nephrostomy; partial obstruction	S (no recurrence)	13	191*
D	SUB#4	Unilateral, left	Left cystostomy; complete obstruction	F	8	3*
E	SUB#5	Bilateral	Left nephrostomy; complete obstruction	S (two recurrences)	7	0
F	SUB#6	Bilateral	Left nephrostomy; complete obstruction	F	19	0
G	SUB#7	Unilateral, left	Left nephrostomy; complete obstruction	F	15	0
H	SUB#8	Bilateral	Left nephrostomy; partial obstruction	S (no recurrence)	14	216*
	SUB#9	Bilateral	Right nephrostomy; partial obstruction	F	37	0*
I	SUB#10	Bilateral	Left nephrostomy; partial obstruction	S (four recurrences)	10	304*
J	SUB#11	Bilateral	Left nephrostomy; partial obstruction	S (no recurrence)	9	380
K	SUB#12	Bilateral	Left nephrostomy; complete obstruction	S (one recurrence)	11	346
L	SUB#13	Bilateral	Right nephrostomy; partial obstruction	S (one recurrence)	4	203
M	SUB#14	Bilateral	Left nephrostomy; complete obstruction	S (one recurrence)	3	3
	SUB#15	Bilateral	Right nephrostomy; partial obstruction	F	13	7
N	SUB#16	Unilateral Right	Right nephrostomy; partial obstruction	S (no recurrence)	8	2*

* Still alive

S = successfully regained patency; F = failed to regain patency

### SUB device obstruction

Eight cats (8/14; 57.1%) were found to have an obstructed SUB device during a routine scheduled appointment and were doing well at home. Four cats (28.6%) were taken to their scheduled appointment presenting with clinical signs: weight loss (n = 1/4), vomiting (n = 1/4), decreased appetite (n = 3/4), intermittent pollakiuria (n = 1/4), abdominal discomfort (n = 1/4) and increased water intake (n = 1). Two cats (2/14; 14.3%) were presented as an emergency because of anorexia (n = 2/2), vomiting (n = 2/2), pollakiuria (n = 1/2) and perceived discomfort (n = 1/2). Focused urinary tract ultrasound revealed renal pelvic dilation in 15/16 kidneys (range 1.3–7.8 mm; median 2.9 mm), ureteral dilation in 6/16 kidneys (range 2–4.5 mm; median 2.7 mm) and dilated calyces in 8/16 kidneys. Twelve cats (85.7%) had unilateral and two cats (14.3%) had bilateral SUB device obstruction. The left nephrostomy catheter was obstructed in 11 SUB devices (11/16; 68.8%), the right nephrostomy catheter was obstructed in four (4/16; 25%) and the left cystostomy catheter was obstructed in one (1/16; 6.3%). The obstruction was classified as partial in 10 SUB devices (10/16; 62.5%), and complete in six (6/16; 37.5%). At the time of the first episode of obstruction, creatinine was elevated in 11 patients with a median of 223.5 µmol/l (range 106–1439; mean 359.3). Furthermore, 50% of cats with SUB device obstruction showed a significant increase in serum creatinine at the time of obstruction compared to their baseline when not obstructed.

### Concurrent medical conditions

Thirteen cats (13/14; 92.9%) presented an increased ionized calcium over the study period (>1.34 mmol/l). Idiopathic hypercalcemia was diagnosed in 2/13 cats based on results of a parathyroid profile. The other 11/13 cats did not have parathyroid profile run thus the etiology of the hypercalcemia couldn’t be determined. Hypercalcemia had been managed in seven cats (7/13; 53.8%) by switching to a fiber-rich diet and two cats (2/13; 15.4%) received a fiber-rich diet and alendronate (30 mg/cat/week). In four cats (4/13; 30.8%), the ionized hypercalcemia was not treated as it was considered mild (⩽1.4 mmol/l). Following SUB device placement, cats were classified in IRIS stages (based on 3 months postoperative creatinine values). Twelve cats were classified as stage 2 and two cats as stage 3.

Three cats (21.4%) had a positive urine culture prior to SUB device obstruction (*Trueperella abortisuis* in one cat and a hemolytic *Escherichia coli* in two cats). At the time of the first obstructive event, no cat had a positive urine culture. Two cats (14.3%) presented intermittent chronic LUTS following SUB device placement. Five cats (35.7%) had a suspicion of inflammatory bowel disease based on clinical signs and/or ultrasound changes, and prednisolone treatment was administered in 2/5 cats. One cat (7.1%) was hypertensive and treated with amlodipine. Three cats (21.4%) were diagnosed with hypertrophic cardiomyopathy and were treated with atenolol (n = 1/3), clopidogrel and atenolol (n = 1/3) and one received no treatment (n = 1/3).

### tEDTA protocol

In reviewing the medical files, the tEDTA protocol used in this study was more intense than that recommended by Norfolk Medical. As soon as a SUB flush was unsuccessful (no visible bubbles within the pelvis or bladder) and pelvic dilation was noted, hospitalization of the patient was recommended so that two tEDTA SUB flushes could be performed daily until patency was achieved. A total of 16 tEDTA infusion protocols were performed in 14 cats. tEDTA protocols relieved obstruction in 11 SUB devices (68.8%) requiring a median of five infusions to restore patency (range 3–12; mean 6). tEDTA failed to relieve the obstruction in 5/16 SUB devices (31.3%) and in these patients, a median of 15 tEDTA infusions were performed (range 8–37; mean 18). In those that failed to regain full patency, 3/5 (60%) SUB devices were completely obstructed. However, despite complete obstruction, two SUB devices regained patency with the tEDTA protocol (27.3%). Overall, infusions were performed over a median of 6.5 active days (range 3–24; mean 7.8). Once the obstruction was relieved, SUB device patency was reassessed and tEDTA infusions were performed 1 month later, then every 3 months long term.

### Outcomes

The tEDTA infusion protocol was considered successful in 11/16 SUB devices (68.8%). Creatinine and pelvic measurements at the start and end of the protocols were available in 5/11 and 9/11 patients, respectively. The median decrease in creatinine was 229 µmol/l (range 61–1234; mean 392.8) and the median decrease in pelvic size was 1.3 mm (range 0.4–6.8; mean 1.8). Ureteral measurements were not consistently recorded. Five SUB devices (5/16; 31.3%) failed to regain patency, despite tEDTA infusions. The following values were calculated based on four patients, as the values for the fifth were not recorded. In these patients, the creatinine value at the start and the end of the protocol increased by a median of 129 µmol/l (range 46–288; mean 148), and the size of the renal pelvis increased by a median of 1.6 mm (range 0.7–2.2; mean 1.5). In the 11 successful tEDTA infusion protocols, six SUB devices (54.5%) had recurrence of obstruction within a median of 87 days (range 29–346; mean 115.3) following the first tEDTA protocol. At the time of writing, 8/14 cats (57.1%) died or were euthanized and six (42.9%) were alive. Cause of death was unknown in 5/8 cats (62.5%) as no abdominal ultrasound or SUB device flush were performed; however, 3/5 cats had an increased creatinine and a deterioration in their overall health status. The owners declined to continue with tEDTA protocols and elected euthanasia in 2/8 (25%) cats. In one cat (12.5%), cause of death was secondary to migration of the nephrostomy tube with enteric internalization (migration of the SUB device in the gastrointestinal tract). The cat underwent surgery for SUB catheter removal but died in the immediate postoperative period from cardiac arrest. A renal-related cause of death was suspected in 6/8 cats (75%). In the six cats alive at the time of writing, four have continued tEDTA flushes every 3 months and their SUB devices have remained patent. Two cats are undergoing monthly tEDTA infusions due to partial persistent obstruction that worsened with longer flush intervals. In all patients, the tEDTA protocol allowed a median extension time in SUB device patency of 71 days (range 2–380; mean 121).

### Complications: tEDTA protocol

One or more episodes of self-limiting pollakiuria (5/8), hematuria (1/8) or both (2/8) following SUB device infusions were reported in 8/14 patients (57.1%). Clinical signs were seen with the use of 2% tEDTA (2/8) and with 4% tEDTA (6/8). Multiple strategies were employed to prevent side effects from tEDTA infusions during the protocol. In 6/8 cats, gabapentin (5–6.3 mg/kg PO q12h) was administered and successfully controlled LUTS. In 2/8 cats, 4% tEDTA was switched to 2% tEDTA (gabapentin was used at the same time) and clinical signs have resolved. In 2/8 cats, no change was made to the tEDTA protocol.

## Discussion

Few studies have reported on the efficacy of tEDTA in the restoration of patency of urinary tract implants. A recent study reported the resolution of mineralization in eight SUB devices using a standard 2% tEDTA infusion protocol.^13^ tEDTA has been investigated for its potential to induce dissolution of urinary tract stones in people. One study evaluated the effect of continuous infusions of 1% disodium EDTA on rabbit bladders. Histopathology showed diffuse edema of the bladder wall, neutrophil infiltration and necrosis of the uroendothelium. It was suspected that the chelating properties were injurious to the bladder and not the chemical structure or the pH of the solution. Because of its numerous side effects on the bladder wall in humans, rabbits and mice, in vivo stone dissolution with tEDTA was abandoned, and other stone removal techniques were developed, such as extracorporeal shockwave lithotripsy.^5,6,16,17^

The use of tEDTA as a non-antibiotic antimicrobial with antibiofilm properties has helped renew interest in its clinical use. Recently, tEDTA was approved as a flush solution for vascular catheters^9^ and for wound treatment.^10^ Its strong chelating ability makes it an interesting tool to dissolve mineral debris obstructing urinary tract implants. In Canada, 4% tEDTA is the only concentration available and therefore it was chosen to treat patients in this study.

The study protocol was adapted from that described by Norfolk Medical (https://norfolkvetproducts.com/wp-content/uploads/2020/02/EDTA_protocol_MINERALIZATION_2020-01.pdf) and intensified with the goal of providing a larger number of tEDTA infusions during a shorter period of time in order to achieve greater patency and facilitate owner compliance. The protocol was also adapted in response to owner scheduling and financial constraints. The estimated cost chart (Table 3) may have an impact on owner decisions regarding performing in-hospital tEDTA protocols, one-time hospital appointments or surgical replacement of the mineralized SUB device. Even if a complete obstruction of the device was noted, surgical replacement was not immediately recommended for various reasons: the need for general anesthesia in elderly patients, cats with heart disease or other comorbidities and the invasiveness of catheter exchange. The cost of 2–3 tEDTA protocols exceeded the cost of catheter replacement at our hospital.

**Table 3:** Cost chart (Canadian dollars) for various approaches to addressing obstructed subcutaneous ureteral bypass (SUB) device catheters in patients with luminal obstruction

Appointment for regular tEDTA SUB device flush	$150–$400 (depending on additional tests performed)
tEDTA protocol (stable patient)	4–5 days of hospitalization: $500–$550 (2–3 flushes daily, depending on the patient)
tEDTA protocol (clinically ill patient)	$1000–$2000
Surgical replacement of the nephrostomy or cystostomy catheter	$3000–$4000
Initial placement of a unilateral SUB device	$6000–$8000
Initial placement of a bilateral SUB device	$8000–$11,000

This protocol, though more intense, may have positively or negatively affected our outcomes. For example, a greater number of infusions over a shorter period of time may have accelerated dissolution of mineralization; however, shorter time intervals between infusions may have resulted in greater exposure of the uroepithelium to tEDTA. The ideal protocol resulting in optimal dissolution accompanied with the least number of side effects has yet to be determined.

In our study, a total of 16 infusion protocols were performed in 14 cats; tEDTA protocols relieved obstruction in 11 SUB devices (68.8%). Recurrence of obstruction occurred in 6/11 SUB devices within a median of 87 days (range 29–346; mean 115) after the first tEDTA protocol. Although recurrence was common, SUB device patency was restored with continued infusions for a median of 71 days. During this time, surgical catheter exchange was avoided and patients had stable azotemia.

A previous study reported that, following SUB device placement, 54% of cats with obstructive ureterolithiasis will pass their stones and eventually regain patent ureters.^18^ In our study, only cats presenting with an obstructed SUB device and persistent ureteral obstruction were enrolled. The authors were concerned about performing tEDTA infusion protocols in cats with obstructed SUB devices without concurrent ureteral obstruction as debris and partially dissolved nephroliths could be flushed into a patent ureter resulting in obstruction. This occurred anecdotally in two patients not included in this study. However, it could be argued that tEDTA infusion protocols should be performed whenever an obstructed SUB device is diagnosed in order to regain patency and avoid a future obstructive event.

Surprisingly, SUB device obstruction was an incidental finding in 8/14 patients, despite the presence of ureteral obstruction. Clinical signs in our patients (6/14; 42.9%) at presentation were attributed to azotemia and not to the obstruction itself. Regular monitoring of kidney disease, focused urinary tract ultrasounds and SUB device flushes were important in this population of cats and allowed for early detection of SUB device obstruction and ureteral obstruction in as yet asymptomatic cats. It is possible that obstruction of the SUB device occurred gradually, allowing the contralateral kidney to partially compensate, thus explaining the stable creatinine in some obstructed cats.

None of the patients in our study was receiving potassium citrate or hydrochlorothiazide to prevent CaOx stone recurrence. A previous study recommended the use of potassium citrate and hydrochlorothiazide in patients with intraluminal obstruction of their SUB devices.^13^ Potassium citrate chelates oxalate and calcium and increases urinary pH, which may help prevent the formation of CaOx stone.^19,20^ Hydrochlorothiazide decreases calciuresis and may prevent CaOx stone formation.^19,21^ The efficacy of these medications for the prevention of upper urinary tract CaOx stones and urinary implant mineralization in cats has not been established.

Our second objective was to determine the safety and side effects observed in cats undergoing 4% tEDTA infusion protocols. Six cats (6/14; 42.9%) did not show any signs of LUTS throughout the study period. No cat had the infusion protocol stopped owing to LUTS. The observed LUTS were generally mild and self-limiting and consisted of macroscopic hematuria and pollakiuria. Following infusions, the litter box was removed for 1 h to try to keep the tEDTA in the bladder and in contact with the cystostomy tube as long as possible. Contact time of tEDTA with luminal mineralization may increase dissolution. Factors reported to affect the mineral dissolution rate are pH (more efficient when pH >10) and contact surface area.^22^ No precise protocol has been established for mineral dissolution; however, a study reported that 1% EDTA solution with a pH of 7.5 was able to dissolve a 2 mm CaOx stone within 48 h.^22^ It is suspected that the contact time required to dissolve luminal mineralization in cats with SUBs would be shorter as small stones and mineral debris are most often responsible for luminal obstruction (authors’ observation). Our study reported a higher incidence of LUTS than the previous study in which no patient presented LUTS following 2% tEDTA infusion protocols.^13^ The higher incidence of LUTS in our study may be explained by the use of a more concentrated tEDTA solution followed by more frequent infusions; however, one patient showed LUTS with 2% tEDTA but not with 4% tEDTA. It was also difficult, in our study, to determine whether LUTS were secondary to the tEDTA infusions or if they were the result of feline interstitial cystitis and/or the irritation from the presence of the cystostomy catheter or displacement of debris following the flushes.

Upon the first obstructive event, 31.3% of SUB devices failed to regain patency despite tEDTA infusions. One of the reasons that could explain why tEDTA infusions were not successful in relieving all obstructive events may be related to the contact time, as mentioned above. Contact time with EDTA is temporary/short-lived when a SUB infusion is performed. When a catheter is blocked, most of the tEDTA travels down the path of least resistance, which is the patent catheter, and therefore very little tEDTA may actually make it to the site of the obstruction. Administering quick, pulsatile injections while flushing the system may help increase the amount that reaches to the blockage. Further investigations are needed to determine how best to deliver tEDTA to the site of obstruction. The efficacy of some techniques, such as placing the cat in a Trendelenburg position, to allow gravity to help tEDTA move through an obstructed SUB device, or pinching off the patent catheter during flushing to help redirect the flush to the obstructed catheter, were not evaluated in this study. In our study, patency was easier to achieve in SUB devices with partial obstruction.

In people, urinary implants are preferably removed as infection with urease-producing bacteria, biofilm and mineralization of the implants commonly occur.^23–25^ Reduced encrustation has been reported with silicone catheters; however, recent veterinary research has shown a decreased prevalence of mineralization with polyurethane catheters (7.8%) in comparison to silicone catheters (17.5%).^1^ Development of catheters resulting in less crystal adherence could help reduce encrustation.

Approximately half (54.5%) of our cats that underwent a successful first tEDTA infusion protocol had a recurrent obstructive event. Reasons for this likely include incomplete dissolution of mineral debris and/or recurrent obstruction from continued mineral deposition and adherence to the SUB catheters bathed in mineral saturated urine.

This study has a number of limitations, most importantly the small number of cats and its retrospective nature. Owing to its retrospective nature, no control group was available, which could have allowed us to better assess the overall efficacy of the tEDTA infusion protocol and better determine whether LUTS were due to the tEDTA, the infusions or other factors. Focused urinary tract ultrasounds were done each time a tEDTA infusion was performed. Renal pelvic and ureteral measurements were not consistently recorded at each infusion. Owing to financial constraints, serum creatinine could not be measured regularly in all patients during the infusion protocols. Protocols were adapted to owners’ schedules and therefore variations in scheduling occurred. It is possible that LUTS were missed in some cats during the tEDTA protocol as hospitalized patients were not under continuous observation.

## Conclusions

Our study showed that a 4% tEDTA infusion protocol restored patency in cats with luminal obstruction of their SUB devices. This protocol should be considered in cats presenting luminal obstruction of their SUB device once radiographs have ruled out kinking and migration as causes for the obstruction. The authors recommend performing this protocol in cats with signs of ureteral obstruction (dilated renal pelvis, flushes not seen in the bladder and kidney ± associated with a rise in serum creatinine) with the goal of re-establishing patency and thus avoiding surgical catheter exchange. The ideal tEDTA concentration and schedule has yet to be established. Recurrence was common and continued patient monitoring was essential.
